# High Differentiation among Eight Villages in a Secluded Area of Sardinia Revealed by Genome-Wide High Density SNPs Analysis

**DOI:** 10.1371/journal.pone.0004654

**Published:** 2009-02-27

**Authors:** Giorgio Pistis, Ignazio Piras, Nicola Pirastu, Ivana Persico, Alessandro Sassu, Andrea Picciau, Dionigio Prodi, Cristina Fraumene, Evelina Mocci, Maria Teresa Manias, Rossano Atzeni, Massimiliano Cosso, Mario Pirastu, Andrea Angius

**Affiliations:** 1 Istituto di Genetica delle Popolazioni, CNR, Alghero, Sassari, Italy; 2 Shardna Life Sciences, Pula, Cagliari, Italy; Louisiana State University, United States of America

## Abstract

To better design association studies for complex traits in isolated populations it's important to understand how history and isolation moulded the genetic features of different communities. Population isolates should not “a priori” be considered homogeneous, even if the communities are not distant and part of a small region. We studied a particular area of Sardinia called Ogliastra, characterized by the presence of several distinct villages that display different history, immigration events and population size. Cultural and geographic isolation characterized the history of these communities. We determined LD parameters in 8 villages and defined population structure through high density SNPs (about 360 K) on 360 unrelated people (45 selected samples from each village). These isolates showed differences in LD values and LD map length. Five of these villages show high LD values probably due to their reduced population size and extreme isolation. High genetic differentiation among villages was detected. Moreover population structure analysis revealed a high correlation between genetic and geographic distances. Our study indicates that history, geography and biodemography have influenced the genetic features of Ogliastra communities producing differences in LD and population structure. All these data demonstrate that we can consider each village an isolate with specific characteristics. We suggest that, in order to optimize the study design of complex traits, a thorough characterization of genetic features is useful to identify the presence of sub-populations and stratification within genetic isolates.

## Introduction

Recent advances in array technologies have open up the possibility of economic and rapid genotyping of entire cohort of population samples. The availability of millions of single nucleotide polymorphisms (SNPs) provides a highly dense map across the human genome, which can achieve adequate power for investigating genome variants associated to multifactorial diseases [1].

Knowledge of the population structure and LD pattern is essential for study design in order to choose the appropriate approach for gene identification [2], [3].

The identification of genetic variants underlying common human diseases are deeply affected by different population structures even if the research is very carefully designed.

In whole genome association studies, population stratification influences results when allele frequencies differ among subpopulations that are not represented equally among cases and controls. Undetected population structure can mimic the signal of association and can lead to false positives or to missed real effect [4]–[6]. Several studies demonstrate that sampling strategies need to take into account substructures even in relatively homogenous genetic isolates and this is even more relevant in inbred populations [7].

Population substructures have been detected in large population groups [8]–[9], but also in relatively homogeneous geographical and cultural genetic isolates as Iceland, Finland and Jewish Ashkenazi, that cannot be considered to be a single, randomly interbreeding population [7], [10]–[12].

LD genome mapping is an important parameter for the design of association mapping studies [13], [14]. It has been suggested that population isolates, particularly those founded recently, have longer stretches of LD than outbred populations [15]. The interest in utilizing population isolates for LD mapping studies has grown considerably, because increased LD values found in such populations, reduce the number of markers needed decreasing costs and improving statistical analysis [15]. Although isolates show a reduced allelic diversity [4], the presence of long-range LD regions could be useful to identify rare genetic diseases [16] more frequent in these populations.

Not all isolates are equal: marker informativity and extent of LD could vary substantially in different isolated populations [3], [17], [18]. Sardinian population could be considered a genetic homogeneous isolate if one focuses on the founder effect causing monogenic diseases such as β-thalassemia, Wilson, etc. [19], [20]. However, studies of the population genetic structure in different sub-regions of the island identified micro differentiation [21]–[23]. The comparison of small areas or isolates villages always revealed differentiation, while different results were obtained comparing larger areas [24]–[29].

The central-eastern area of Sardinia called Ogliastra, is characterized by the presence of several distinct villages with different history, immigration events and number of inhabitants [21], [30]. Centenarian cultural isolation and conservatism characterized the history of these communities [31]. The relevant features of villages in this geographic area are similar environmental conditions, high endogamy, low immigration and remote origin [28], [32]. This region also appears genetically differentiated from neighboring areas [21].

We focused our analysis on eight Ogliastra isolated villages using a wide set of about 360000 informative SNPs to determine the extend of genetic variation in this population. Other papers compared genetic features in isolated populations but they either used a limited number of SNPs or single chromosomes or have referred only to partial regions [1], [15], [33]. On the contrary, we wanted to examine population structure in all eight villages using a extensive subset of SNPs covering the entire genome.

Our aim was to understand how genetic features can be influenced by history and by geographical and cultural isolation in different communities in order to better determine the feasibility and design of genetic analysis for complex traits.

## Materials and Methods

### Sample characteristics

**Figure 1:**
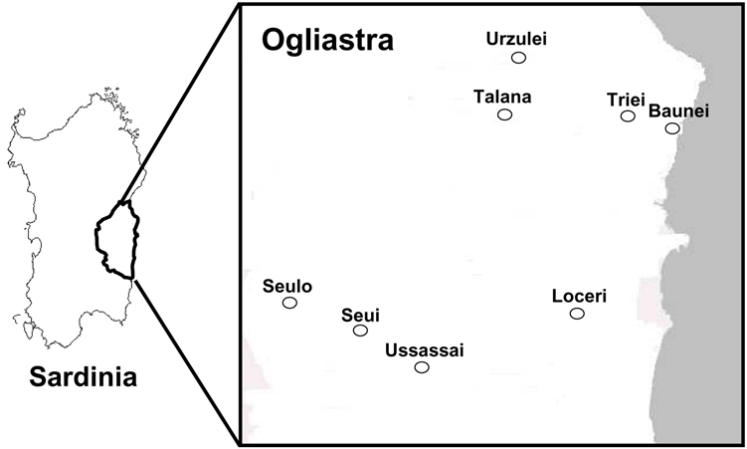
On the left, geographic localization of Ogliastra region in Sardinia. On the right, detailed location of the 8 villages analyzed.

Historic and demographic data demonstrate that Sardinia experienced waves of successive invading populations that pushed the original inhabitants into the most remote and inaccessible areas. The central-eastern Sardinia region, identified as Ogliastra, is clearly one of these refuge areas, geographically and socially secluded for thousands of years from other Sardinia regions due to mountains and deep river valleys. Only after the Second World War, emigration and economic opportunities have changed the population structure of some of these mountain villages leaving in place mostly the older generations of people who still reflect the high level of consanguinity of past inbreeding and who still follow the traditional life styles. To conduct our research we recruited about 9000 voluntary subjects in 8 selected villages: Talana, Urzulei, Baunei, Triei, Seui, Seulo, Ussassai and Loceri (figure 1). All are in the Ogliastra region except for Seulo (which is in Barbagia, another refuge area of Sardinia) but only 5 kilometers from Seui. Moreover, Seulo was in the past included in the Bishopric of Ogliastra and all of its parish registers (*Quinque Libri*) are also conserved in the same archive.

**Figure 2:**
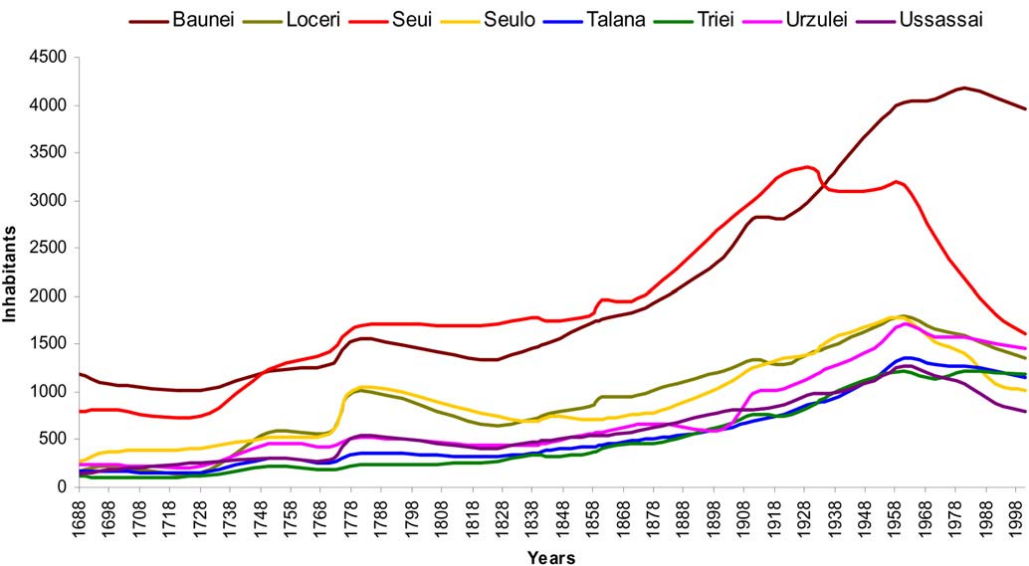
Population growth in the 8 villages analyzed starting from the 17^th^ century to the year 2001.

The 8 selected communities share similar demographic features: limited number of founders, high endogamy and consanguinity. We reconstructed their demographic dynamics over the centuries in order to assess their degree of isolation and inbreeding. Six small villages showed similar population growth trend from 1688 to 2001. Baunei and Seui differ because they were two large villages: Seui was one of the most important centers from the 17th century to 1931 because of anthracite and copper mines but today, because of emigration toward Cagliari, is comparable in size to the other small villages (figure 2).

**Figure 3:**
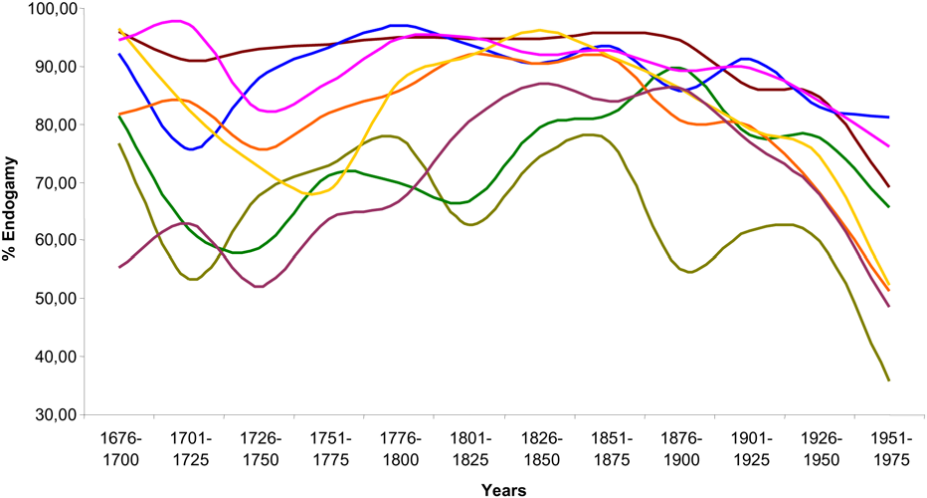
Endogamy (values in %) in the Ogliastra villages from 1676 to 1975.

We calculated the level of endogamy characterizing these populations from 1676 to 1975 (figure 3). Endogamy was calculated as the percentage of marriages' number among people from the same village divided by the marriages celebrated in the same 25 years interval [34].

The endogamy reaches a very high degree in each village from 1676 to 1950 with an average of 80.75% [min 67.18% (Loceri); max 92.72% (Baunei)], starting a slow progressive decrease from 1900 to 1950. From 1950, we observe a severe endogamy decline in all villages (average 57.03%) except for Talana (81.28%).

We reconstructed for each village extensive genealogies based on a relational database that includes all the inhabitants personal data starting from the 17^th^ century. Based on kinship coefficients [35], we selected 45 unrelated individuals from each of the 8 villages forming a sample of 360 individuals. All selected individuals descent from families that were born and lived in the same village for at least four generations. Moreover, as comparison, we also subsequently carried out the same type of analysis (LDU, D' and r^2^ estimates) on 43 individuals originating from other geographical areas of Sardinia.

People living in the village were invited to take part in the study by invitations sent by mail to every family and through public announcements. All the people participating in the study were healthy individuals and volunteers. The personal data were encrypted and kept separated from genetic and data. The scientific content of the project was extensively explained to all individuals participating in the study that signed informed consent forms in accordance with the Helsinki Declaration. The study was submitted and approved by the Italian Ministry of University and Research (MIUR) following the current Italian legislation.

### Genetic Analysis

#### SNP genotyping and quality control

Genomic DNA was extracted from 7 ml of EDTA-treated blood with standard methods. SNPs genotyping for all samples was performed using the Affymetrix GeneChip platform. We utilized the GeneChip® Human Mapping 500 K Array Set that comprises two arrays (the Nsp and Sty arrays) capable of genotyping ∼262000 and ∼238000 SNPs, respectively. We followed the recommended protocol described in the Affymetrix manual. All DNA samples were normalized to 50 ng/µl. Then, 5 µl (250 ng) of dsDNA was digested with the appropriate restriction enzyme and ligated to adapters using T4 DNA ligase. Samples were then PCR amplified using TITANIUM Taq polymerase on an GeneAmp® PCR System 9700 gold plate thermal cycler. PCR products were purified using the Clontech purification kit followed by fragmentation. Samples were then injected into cartridges, hybridized, washed and stained. Mapping array images were obtained using the GeneChip Scanner 3000 7G plus. For quality control (QC), individual arrays not passing the 93% call rate threshold at P = 0.33 with the Dynamic Model algorithm [36] were considered a failure and re-genotyped. The average QC call rate of the data produced using the Nsp arrays was 95.25% while the Sty array reached to 95.56%. All individuals passing the QC quality checks were further screened based on genotyping performance. Genotypes were called using the BRLMM (Bayesian Robust Linear Model with Mahalanobis distance classifier) software [37]. The average call rate of entire sample was 98.66% and 98.82% for Nsp and Sty fractions respectively. Any individual with genotyping call rates less than 95% for either Nsp or Sty fractions were excluded. All individuals with call rates >95% showed an average of 91.07% SNPs call rate. In addition, individuals whose gender call from X chromosome genotype data was discrepant with the gender obtained from medical records were excluded from the analysis.

All SNPs physical coordinates refer to the NCBI released annotation update for the human genome (NCBI Build 36.2). Alleles are expressed in the forward (+) strand of the reference.

The mean minor allele frequencies (MAF) in our isolated population using the 500 K Array GeneChip® showed an average of 0.187 (±0.003). After the exclusion of monomorphic SNPs, the MAF increased an average of 0.251 (±0.004). We selected only SNPs informative (MAF>0.01) in all villages obtaining a sub-sample of 361980 SNPs (average space 7672 bp).

#### Linkage Disequilibrium Maps and Pair-wise LD metrics

LDMAP program [38] was used to construct LD maps. The LD map describes the fine variations in LD pattern over a given chromosomal segment, calculating linkage disequilibrium units (LDU) between adjacent pairs of SNPs. The LDU scale is constructed from the product of physical (kb) distance and a parameter describing the exponential decline in association with distance computed for each interval between adjacent SNPs. In the LD maps, the regions with high LD can be identified as plateaus where the increase in LDU is very small or zero, while the regions with lower LD can be identified as steps. The LD maps were calculated on autosomes based on all 361980 SNPs for the 360 samples. Thus, comparison of LD maps in the villages was based on the same SNPs and on the same number of samples.

Two types of pair-wise LD metrics were calculated: Lewontin's standardized deviation coefficient |D'| [39] and the pair-wise correlation r^2^ [40] between all pairs of SNPs within 500 Kb of each other, using the program Haploview [41] (http://www.broad.mit.edu/haploview).

### Structure and population analysis

#### Single nucleotide polymorphisms (SNPs) analysis

For population analysis, 500 K were filtered according to inter-SNP distance (about 500 kb) to minimize LD effects, obtaining 5,262 SNPs. We tested Hardy–Weinberg equilibrium with a modified version of the Markov-chain random walk algorithm described in Guo and Thompson [42]. The modified version gives the same results as the original one, but is more efficient from a computational point of view. The analysis was performed with the software Arlequin 3.1 [43], (http://cmpg.unibe.ch/software/arlequin3/). Seventy markers were excluded from the following analysis, as they showed highly significant departure from Hardy Weinberg equilibrium (P<10^−5^), obtaining 5,192 markers for the following analysis.

To prepare haplotype data, we considered 83 genomic regions with D'>0.7 in all 8 communities, starting from data employed for LD analysis, for a total of 361980 SNPs. Since recombination in X-linked and autosomal portions of the human genome is often concentrated in hotspots separated by DNA regions with little or no recombination [44], [45], we felt that our choice of high LD genomic regions (precisely D'>0.7) in our samples would be the most informative.

We estimated haplotype frequencies using windows of 10 consecutive informative SNPs for each region, spanning an average of 150 kb.

Inference of haplotype frequencies were performed employing the software PHASE 2.1 [46], [47].

Haplotype data were used to compute genetic distances. Each region was considered as a single polymorphic locus, where each haplotype corresponded to a single allele.

F_ST_, a standard measure of genetic differentiation between populations [48] was estimated with the Weir and Cockerham algorithm [49]. F_ST_ is the proportion of the total genetic variance contained in a subpopulation relative to the total genetic variance. Values can range from 0 to 1, and a high value implies a considerable degree of differentiation among populations. The 95% confidence limits for F_ST_ were determined by 1000 permutations test. Level of statistical significance was tested by performing 1000 permutations. Both analysis were performed with GENETIX 4.5.02 software [50], (http://www.genetix.univmontp2.fr/genetix/genetix.htm).

To ascertain the proportions of genetic variance due to differences within and between populations, genetic variance was hierarchically apportioned according to geographic criteria through the locus by locus AMOVA (analysis of molecular variance) [51], using the Arlequin 3.01 software [43]. Number of permutations was set at 10000.

Genetic distances were computed from allele and haplotype frequencies according to Nei [52] and Reynolds [53], with the PHYLIP v 3.66 [54] executable “Gendist”, after processing of dataset for bootstrapping (1000 replicates), using PHYLIP executable “Seqboot”. We applied two different measures of genetic distances to test different assumptions. All assume that all differences between populations arise from genetic drift. Nei's [52] distance model, is based on infinite isoallele model of mutations [55], where all loci have the same rate of neutral mutations, and that the genetic variability originally in the population is at equilibrium between mutations and genetic drift. Moreover, the effective population size of each population remains constant. Reynolds' distance [53] assumes that there is no mutation, so that gene frequency changes are by genetic drift alone. In addition, population sizes do not remained constant and equal in all populations.

Distances were clustered using the Neighbor – Joining method [56], and consensus tree were built with the PHYLIP executable “Consense”. We used the Neighbor Joining method instead of other methods as UPGMA (Unweighted Pair Group Method of Analysis) or ML (Maximun Likelihood) because it was shown to be generally better than the other methods [56]. Phylip package is available at the following link: http://evolution.genetics.washington.edu/phylip.html.

We performed factor Correspondence Analysis (FCA) using the Genetix 4.5.02 software [50]. FCA [57]–[59] is a kind of canonical analysis particularly well suited to describe associations between two qualitative variables (analysis of a contingency table crossing the terms of two variables). She et al. [60] have proposed a more appropriate coding of data for the genetics of diploid organisms, whereas others authors [61]–[63] define the correlation between the results of the analysis and conventional parameters of population genetics.

Details of mathematical principles of methods are provided at the following link: http://www.unesco.org/webworld/idams/advguide/Chapt6_5.htm. We used this method because Guinand [63] indicated that CRT-MTC (whose FCA implemented in Genetix utilize a similar strategy) presents various advantages in respect to PCA (Principal Component Analysis) in estimating some parameters and in the presence of a pooling strategy. Correspondence analysis has been used recently with good results in the Seldin et al. study [8]

We examined correlation between genetic and geographic distance matrices using the Mantel test by a permutation procedure. The permutation allows to examine the empirical null distribution of the correlation coefficient taking into account the auto-correlations of the elements of the matrix [64], [65]. Here, we compared genetic distance computed with the described method obtaining a null distribution with 500000 permutations. The analysis was performed with Arlequin 3.1 software [43].

To infer individual ancestry and population admixture, we carried out analysis of population structure using a model-based clustering method developed in the STRUCTURE 2.2 software [66] (http://pritch.bsd.uchicago.edu/structure.html). The model assumes the presence of K populations (where K may be unknown), each of which is characterized by a set of allele frequencies at each locus. Individuals in the sample are assigned probabilistically to one cluster or to two or more clusters if their genotypes indicate that they are admixed [67]. We conducted the analysis according to the admixture model and without any prior population assignment. We performed several runs for each K (from 2 to 8), until we obtained 10 times the same result, using 20000 replicates and 10000 burn-in cycles.

## Results

### Measuring Linkage Disequilibrium

We compared the extent of LD for the 8 villages and for a sample originating from other geographical areas of Sardinia. We used a set comprising both commons and informative SNPs in all communities. Moreover, to compare our results to a standard outbred population, we included in our study 60 unrelated samples from 30 HapMap CEU trios. The mean heterozygosity of the markers was 0.345 (SD = 0.011) in the 10 communities selected, ranging from 0.320 (Urzulei) to 0.361 (Seui). The average of minor allele frequency (MAF) was 0.251 (SD = 0.004), ranging from 0.241 (Urzulei) to 0.257 (Seui).

The monomorphic SNPs (M-SNPs), in at least one village, were ∼26% of the entire SNPs list. The shared M-SNPs in the eight villages were 31656. The comparison with the HapMap samples showed that 88.5% of the shared M-SNPs were monomorphic also in the CEU population and 10.89% showed a MAF>0 and <0.05. Considering the total number of M-SNPs in each village, we identified a group of village specific M-SNPs that range from the highest values in Urzulei and Talana (5.04% and 4.33% respectively) to the lowest in Seui and Loceri (1.85% and 1.45% respectively).

**Figure 4:**
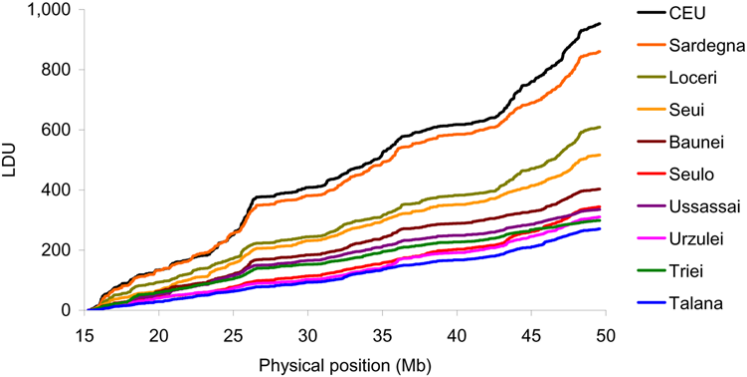
Comparison of population-specific LD maps on chromosome 22 of the 10 samples from Ogliastra villages, rest of Sardinia and CEU.

We constructed LD maps for the 10 populations on autosomes. The shortest maps were in Talana, Urzulei, Ussassai, Triei and Seulo, in agreement with their small population size and their secluded position. We found the trend growing from Baunei to Seui and to Loceri. The longest maps were in the Sardinia and in the CEU samples (figure 4). Similar results and differences in LD maps were highlighted in all chromosomes analyzed. Moreover the metric LD map evidence a pattern of “plateaus”, regions of high LD, and “steps”, regions of increased recombination: this pattern tends to be shared among all villages. In addition, the comparison with CEU population confirmed this pattern and presumably reflects the common distribution of recombination hot spots across various populations [68].

**Figure 5:**
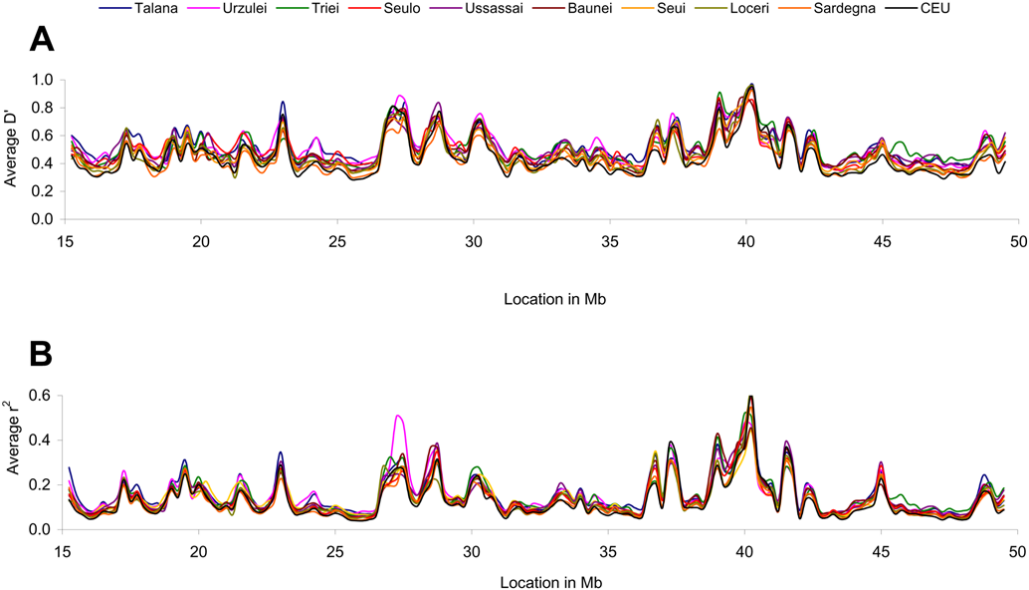
Distribution of Linkage Disequilibrium on chromosome 22. Average D' (A) and r^2^ (B) coefficients plotted in 0.5 Megabases sliding windows (0.25 Mb overlap).

We compared the extent of LD by examining the distribution of D' and r^2^ on autosomes in the selected communities. We calculated D' and r^2^ between all pairs of markers (figure 5). The highest D' and r^2^ average values were detected in Talana, Urzulei, Ussassai, Triei and Seulo, and the lowest in Sardinia and CEU.

In performing LD analysis in small populations it is important to consider the kinship value among the subjects, because most individuals are in some way related to each other. This relatedness may inflate estimates of LD. Utilizing the genealogical information present in our database, we reconstructed all the family relations for each individual in each villages and calculated the pair wise relatedness of individuals. We selected 45 unrelated individuals from each village reaching a total of 360 subjects (table 1). The average kinship value goes from the highest value in Talana (0.021) to the lowest in Loceri and Baunei (0.004). To evaluate how the degree of relatedness influences LD, we analyzed the LD pattern on 10 randomly chosen groups of 45 individuals each from Talana. The average kinship of these 10 groups is comprised between 0.022 and 0.025. The LD map length of the randomly chosen groups ranged from 292 to 288 LDU.

**Table 1:** For each village we report resident population size, average village kinship values, number of samples and its average kinship values.

	Today population	Average Village kinship	Samples	Average Sample Kinship
**Talana**	1129	0.024	45	0.021
**Urzulei**	1443	0.017	45	0.013
**Baunei**	3886	0.006	45	0.004
**Triei**	1115	0.020	45	0.016
**Seulo**	1023	0.016	45	0.014
**Seui**	1587	0.007	45	0.005
**Ussassai**	763	0.017	45	0.012
**Loceri**	1336	0.010	45	0.004

### Population structure analysis

In order to assess the level of differentiation among the villages we calculated the F_ST_ values by the Weir and Cockerham [49] algorithm (table 2). F_ST_ for the 8 communities was 0.0213. We found the highest difference between the villages of Talana and Seulo (F_ST_ = 0.0324) and the lowest difference between Baunei and Triei (F_ST_ = 0.0105). All the comparisons between villages were highly significant (P<0.0001).

**Table 2:** Fst values computer for each pair-wise comparison calculated on 5262 SNPs evenly spaced on 500 Kb. Corresponding 95% confidence intervals, shown between parentheses, were determined with permutations testing (set at 1000).

	*Baunei*	*Loceri*	*Seui*	*Seulo*	*Talana*	*Triei*	*Urzulei*
***Loceri***	0.01563 (0.01462–0.01664)						
***Seui***	0.01970 (0.01843–0.02096)	0.01089 (0.01007–0.01172)					
***Seulo***	0.02520 (0.02396–0.02649)	0.01724 (0.01628–0.01836)	0.01435 (0.01335–0.01536)				
***Talana***	0.02296 (0.02166–0.02442)	0.02197 (0.02081–0.02319)	0.02590 (0.02447–0.02727)	0.03238 (0.03061–0.03404)			
***Triei***	0.01051 (0.00971–0.01136)	0.01854 (0.01737–0.01971)	0.02245 (0.02112–0.02371)	0.02883 (0.02734–0.03033)	0.02500 (0.02361–0.02643)		
***Urzulei***	0.01719 (0.01607–0.01831)	0.01769 (0.01651–0.01871)	0.02312 (0.02172–0.02444)	0.02942 (0.02790–0.03090)	0.02346 (0.02213–0.02482)	0.02051 (0.01923–0.02174)	
***Ussassai***	0.02524 (0.02395–0.02664)	0.01435 (0.01335–0.01536)	0.01096 (0.01009–0.01187)	0.01938 (0.01813–0.02060)	0.03051 (0.02894–0.03210)	0.02766 (0.02616–0.02923)	0.02728 (0.02577–0.02863)

The levels of statistical significance were tested by performing 1000 permutations. All comparisons were highly significant (P<10^−3^).

For each village, we computed average Fst resulting from each pairwise comparison. We observed the highest degree of differentiation for Talana and Seulo villages, with F_ST_ = 0.0260 and F_ST_ = 0.0238, respectively. Moreover, these two villages show a great deal of genetic distance between each other (F_ST_ = 0.0324).

When we computed F_ST_ values pooling villages according to geographic position (Talana, Urzulei, Baunei, Triei in the first group and the others villages in the second), we observed a higher F_ST_ value in the first group (F_ST_ = 0.0200) than in the second (F_ST_ = 0.0142).

For AMOVA analysis [51], we grouped villages in two groups according to geographic position, as in the previous F_ST_ analysis. Results reveal a statistically significant genetic heterogeneity between the two geographic areas (F_CT_) (0.75% of total variation, P<10^−5^). Also the heterogeneity among villages within areas (F_SC_) and among individuals within villages (F_ST_) is highly significant (1.69%, P<10^−5^ and 97.56%, P<10^−5^).

**Figure 6:**
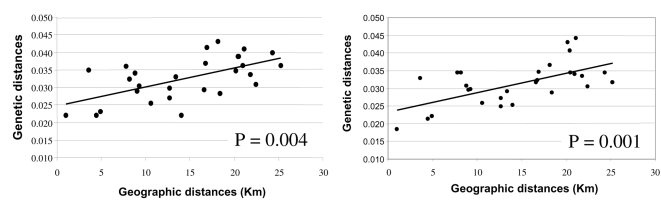
Genetic distance as a function of geographic distance between each pair of communities. On the left (A) Mantel Test plot analysis using SNPs data; on the right (B) Mantel Test plot analysis using Haplotype data.

Population structure was analyzed with the mean of the Mantel Test, which compares coefficient correlation between geographic and genetic distances [64], [65]. Also in this case, we performed the analysis using both Nei [52] and Reynolds [53] distances. We observed in both SNPs and haplotype analysis, a significant correlation (P = 0.004 and P = 0.001), indicating a pattern of isolation by distance in the Ogliastra genetic pool (figure 6A and 6B).

**Figure 7:**
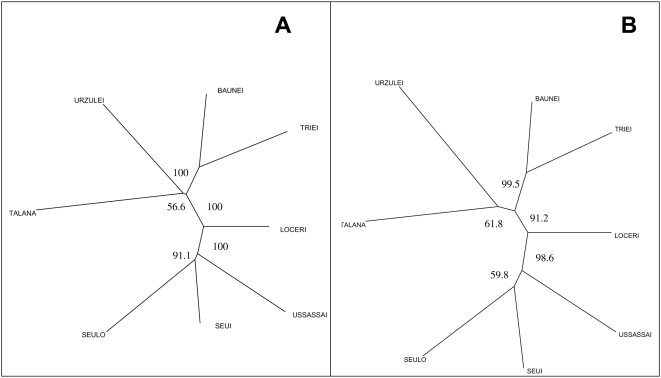
Neighbor Joining trees analysis. A) Neighbor Joining tree computed with Reynolds (1983) distances from allele frequencies of 5192 SNPs. Bootstrap supports over 50% (out of 1000 iterations) along the nodes. B): Neighbor Joining tree computed with Reynolds (1983) distances from haplotype frequencies of 83 regions containing 10 SNPs. Bootstrap supports over 50% (out of 1000 iterations) along the nodes.

Figure 7 shows the Neighbor Joining tree computed with Reynolds [53] genetic distances from allele (7A) and haplotype frequencies (7B). We performed the same analysis with Nei [52] distances, with identical results (data not shown). The tree topology corresponds to the geographic distribution of the two cluster areas in the Ogliastra region with the village of Loceri in an intermediate position.

**Figure 8:**
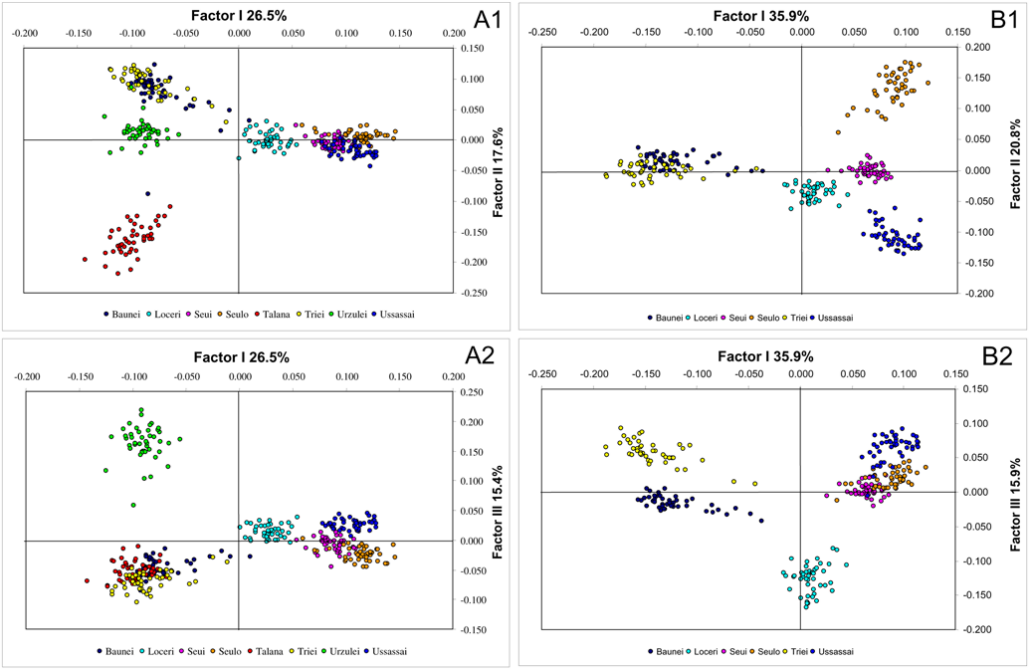
Factor Correspondence Analysis comparing different individuals from different Ogliastra villages, performed with 5,192 SNPs. Each individual is represented by a circular shape, and the different 8 communities are marked with different colours. The two plots represented factor 1 and 2 and factor 1 and 3. (A1, A2) Analysis performed with 8 communities; (B1, B2) Analysis performed with 6 communities, without Talana and Urzulei.

We plotted results of Factor Correspondence Analysis [63] performed with allele frequencies of the 8 communities for factors 1 and 2 (26.5% and 17.6% of inertia, respectively) and for factors 1 and 3 (26.5% and 15.4%, respectively). Results confirm differentiation between the two geographic areas (figure 8A1–8A2), separated by factor 1, highlight the strong differentiation of Talana-Urzulei versus other villages (factor 2), and of Talana versus Urzulei (factor 3). We repeated the computation excluding Talana and Urzulei, to better visualize patterns of population relationships among the remaining six villages, (figure 8B1–8B2). Factor 1 accounts for 35.9% of inertia, and factor 2 and 3 represented 20.8% and 15.9% of inertia. This analysis confirms the differentiation in two main regions, separated by factor 1. Factor 2 highlights the intermediate position of Seui between Seulo and Ussassai, and factor 3 suggests a notable differentiation between Baunei and Triei.

**Figure 9:**
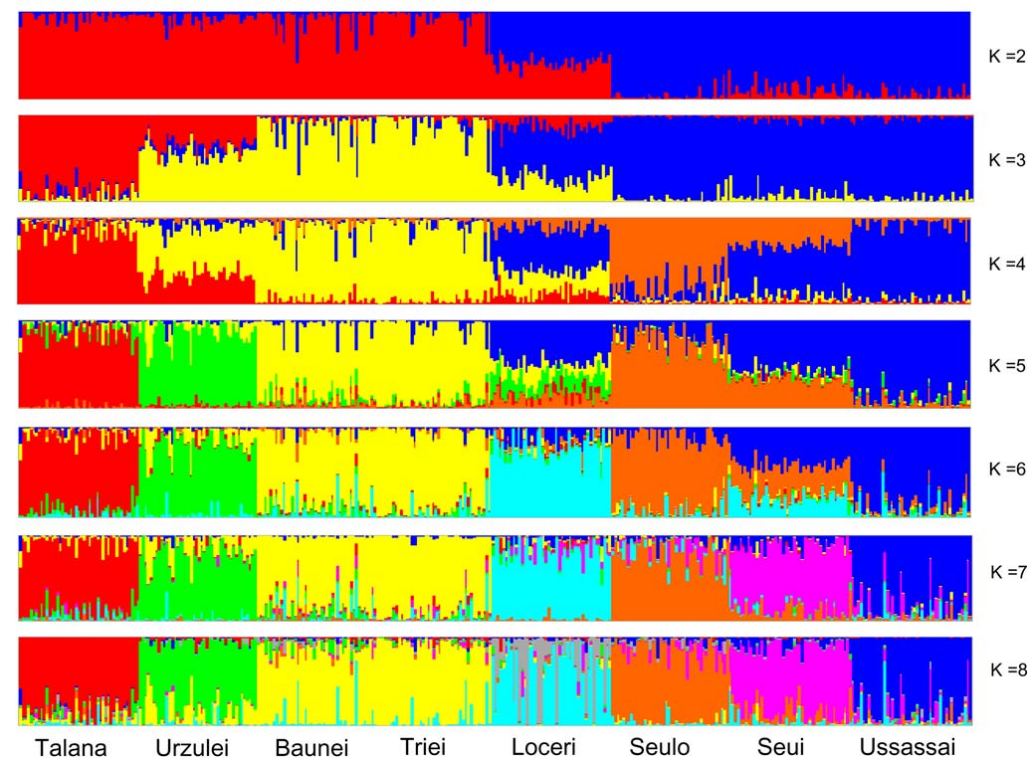
STRUCTURE results under the assumption of different population groups (K  =  2:8). Each individual is shown as a vertical line partitioned into K colored components representing inferred membership in K genetic clusters. K  =  7 represents the highest values of lnP(D).

To performed STRUCTURE analysis [66], we assessed number of population groups (K), performing 10 runs at each K, from 2 to 8. The estimation of log_e_ probability of the data using the F model favored the assumption of K = 7. Figure 9 shows results at different K values. Analysis at K = 2 illustrates the differences of the two regions, while at subsequent K values we noted the differentiation of Talana, Urzulei, Baunei and Triei in comparison with southern Ogliastra. The remaining villages are included at subsequent K values. At K = 8, we detect 8 different clusters which do not correspond to each single village: Triei and Baunei were in the same cluster while Loceri is composed by two different clusters.

## Discussion

We analyzed features and different aspects of population structure in eight villages of Ogliastra region in the central-eastern area of Sardinia. This region is characterized by high endogamy, low immigration, environmental homogeneity, as well as genetic differentiation from the rest of the island [21]. Gene diversity measures of HVSI mtDNA haplotypes suggest that Ogliastra ranks among the most genetically homogeneous European populations [23]. Furthermore, Ogliastra has lowest values of mtDNA gene diversity respect to other areas of Sardinia [23], [27], [69]. A more precise analysis of distinct sub-populations in the same region, revealed a striking differentiation due to distinctive founder effects and genetic drift. This was confirmed by our studies of mtDNA and Y chromosome haplotypes on few Ogliastra villages [27], [32], [70]. Such differentiation was hinted at by our previous LD analysis with microsatellite markers in the Xq13.3 region [17].

In this paper we focus our analysis on eight isolated Ogliastra villages to evaluate LD parameters and effective population structure using a dense whole genome SNPs map. The length of LD map is inversely related to the extent of LD over a given chromosomal segment, therefore shorter LD maps are observed in population isolates compared with more heterogeneous populations. Although the geographic area analyzed in this study was relatively restricted, our results evidence high values of LD parameters and the presence of LD variability across the genome in these communities depending on their historical demography and population structure. The 5 smallest and secluded villages show the shortest LD maps, in agreement with their similar size and isolated condition: from 270 LDU in Talana to 343 LDU in Seulo. We identified intermediate values in Baunei (LDU = 402), a large community with a continuous growth from the 17^th^ century to today.

Seui, on the other hand, has peculiar historical and demographic aspects. This village, which was already an important administrative centre during the Spanish domination of Sardinia (even has a prison dating from the 1640s) has undergone immigration waves starting from the mid-19^th^ century due to the presence of anthracite and copper mines. During the 19^th^ century, Seui became one of Ogliastra main economical centre and the population grew to about 3000 inhabitants from the beginning of the 20^th^ century to the 1960s. Then there was a massive emigration wave toward the main political and economic centre of Sardinia (Cagliari) with a rapid decrease of resident population. Today there are only 1587 inhabitants, and this has influenced LD parameters and current population structure.

Finally, Loceri has the lowest LD values because it is not in an isolated geographical position, it is close to the seashore and to Lanusei, an important Ogliastra administrative centre.

Its frequent genetic exchanges with other villages are proven by archival data spanning many centuries.

Additionally, the extreme features of some villages such as Talana and Urzulei, were evidenced by the analysis of the monomorphic SNPs. The different percentage of specific monomorphic SNPs shows a trend similar to that of different populations, it is consistent with our analyses of LD values and it contributes to evidence the different level of isolation of these villages.

The STRUCTURE population analysis confirms the presence of a distinct cluster for each village analyzed, except for Baunei and Triei (which in the past were part of a single municipality). Moreover, we observed high values of F_ST_ among villages. Recently various studies have analyzed comparable set of data obtained from European and East Asian Populations [71], [72]. We compared our results with Europeans. In the analysis of Italian, Swedish and Spanish cohorts [8], the total value of F_ST_ from a subset of 5,700 SNPs distributed over entire genome, computed with the same method (F_ST_ = 0.0029) was lower than our sample (F_ST_ = 0.0213). The highest degree of differentiation has been detected between samples from Italy and Sweden (F_ST_ = 0.0060). This value is lower than the one detected between Baunei and Triei (F_ST_ = 0.0105), which are the least differentiated villages in this study. Salmela et al. [72] analyzed the structure in various North Europe populations (Finland, Sweden, Northern Germany and Great Britain) and computed F_ST_ values in a subset of 6369 SNPs, finding values lower (from 0.0005 to 0.0072) than values observed among Ogliastra villages (0.0105 to 0.0324).

Our data suggest a great level of differentiation among Ogliastra villages: even if this result is influenced by the difficulty in detecting micro differentiation in countrywide samples, nevertheless it clearly reveals the high level of population substructure in Ogliastra.

In general, our results show a good correlation with the geographical structure of the region, as revealed by the pattern of isolation by distance of the Mantel Test. In particular, Neighbor Joining trees computed from different sets of data (single SNPs and haplotypes) confirmed the Mantel Test results because villages are located in the tree according to their geographic position and relative closeness. Furthermore, the topology of the tree shows two main clusters identifying a Northern and a Southern Ogliastra sub-area. This is confirmed by AMOVA and STRUCTURE analysis at low level of differentiation (K = 2), highlighting the separation of the two sub-areas.

Factor Correspondence Analysis and STRUCTURE analysis emphasize that Talana and Urzulei are highly differentiated although separated by limited geographic distance. In addition, Neighbor Joining tree shows the village of Loceri to be in an intermediate position, while correspondence analysis confirms that Triei and Baunei are highly correlated but not identical.

Our results, obtained with a large SNPs data set, confirm a high degree of differentiation among villages, leading us to some considerations: not only small areas as Ogliastra should not be considered homogeneous a priori, but even adjacent villages could be not homogeneous.

A number of analyses performed in the large and small genetic isolates showed either the presence or absence of substructure. For example, in Hutterites communities we cannot identify distinct subpopulation [73]. Alternatively, the analysis of the Icelandic genetic pool, carried out with 40 microsatellite markers in 43748 individuals from 11 different geographic regions of the island, showed the presence of notable regional subdivisions. Icelanders cannot be considered to be a single randomly interbreeding population [7]. Similarly, the analysis of 14 biallelic markers and 8 STRs of Y-chromosome on samples from different areas of Finland revealed the existence of a sharp genetic border between eastern and western Finland [11], confirmed also by epidemiological differences [74], [75]. Furthermore, a recent analysis with genome wide SNP data, demonstrate the presence of substructure among 10 distinct Finnish early and late-settlement subpopulations [10].

In the case of small population isolates, the analysis of 6 South Tyrolean villages performed with microsatellites on Xq13.3, showed the presence of three genetically distinct sub-populations, sharing the alpine environment and lifestyles [76]. Vitart et al. [77] found genetic differentiation among 10 small isolated villages in Croatia islands. Estimated F_ST_ values were 0.02 (95% CI: 0.017–0.022), with many significant differences in the comparison of these villages.

The information obtained on the genetic differences present among our villages in Ogliastra is potentially useful for the ongoing extensive genetic research in this specific population. Detailed knowledge of the high differentiation in the degree of LD background found in each village will probably be crucial in the analysis of these sub-isolates that represent useful populations for the initial detection of loci/genes with Genome-wide association (GWA) involved in the predisposition to complex traits. Such analysis require a great deal of attention to exclude false positive results as consequence of stratification differences between cases and controls [78]–[80]. Association studies can be confounded by differences in ancestry: geographic ancestry can explain just a portion of human genetic variation. Several genomic variants had high frequencies in populations e/o cohort of samples with particular ancestries and such variants could erroneously appear to be related to disease. These incorrect results can be attributed in part to complex relationships reflecting different population origins that included migration, admixture and isolation.

There are potential advantages in the choice of these populations because of reduced environmental complexity and a probably reduced number of disease alleles. Some of the loci are expected to have stronger genetic effects on the disease/trait under analysis. Furthermore, small isolates might contain mutations e/o possible variants that are rare in the population at large, thus allowing the identification of genes that would otherwise be missed [81].

One negative aspect could be the limited number of cases in association studies, except for common diseases with high prevalence. In fact our group was able to identify a variant related to the EDA2R gene strongly associated with Androgenetic Alopecia showing an average prevalence of 47% in the 8 villages [82].

We also propose that this kind of populations is helpful in the search for variants associated to quantitative traits (QT) related to common disease. In QT loci analyses all individuals phenotyped are useful and even the limited number of inhabitants of one of our villages could be a sufficient cohort for initial association studies. Once a significant association is obtained we can replicate the findings in the other villages. Obviously, it will be important to replicate the findings in a general Sardinian sample and in outbreed populations.

To conclude, we underline the influence of history and bio-demography on the genetic features of population isolates and we propose as a first step, the genetic characterization of isolates in order to identify possible sub-populations and stratification. This could lead to the optimization of study design for the choice of the best approach for gene identification in complex traits.
